# Prospective orofacial quantitative sensory testing data of the human face and mouth

**DOI:** 10.1016/j.dib.2023.109316

**Published:** 2023-06-14

**Authors:** Julie Adriaenssens, Hannah Vaesen Bentein, Reinhilde Jacobs, Constantinus Politis, Fréderic Van der Cruyssen

**Affiliations:** a Department of Oral & Maxillofacial Surgery, University Hospitals Leuven, Leuven, Belgium; b OMFS-IMPATH Research Group, Department of Imaging and Pathology, Faculty of Medicine, University Leuven, Leuven, Belgium; c Department of Oral Health Sciences, KU Leuven and Department of Dentistry, University Hospitals, Leuven, Belgium; d Department of Dental Medicine, Karolinksa Institutet, Stockholm, Sweden

**Keywords:** Oral, Facial, Neurosensory function, Quantitative sensory testing, QST, Trigeminal nerve

## Abstract

Quantitative sensory testing (QST) is a valuable tool in the assessment of orofacial somatosensory function and dysfunction. QST is a method where thermal and mechanical stimuli are applied to the area of interest in a noninvasive way. The QST technique can detect patterns of loss of sensation that may happen in case of hypoesthesia, hypoalgesia, anesthesia, or gain of sensation in the context of allodynia, hyperalgesia or spontaneous pain. Normal values have already been recorded for some parts of the face and mouth, but not for the complete innervation area of the trigeminal nerve. This dataset involves orofacial QST gathered from ten healthy volunteers, a standardized QST battery was applied to 24 regions (14 extraoral and 10 intraoral) innervated by the trigeminal nerve. Descriptive statistics were applied to compare the different regions. This dataset can be used to inform future studies involving orofacial sensory function, pain studies and pharmacological trials.

**Specifications Table**

**Table:** 

Subject	Dentistry, Oral Surgery and Medicine
Specific subject area	Human orofacial somatosensory function using quantitative sensory testing.
Type of data	Table Chart Graph Figure
How the data were acquired	- For all tests the participants were seated in an examination chair, asked not to look at the computer screen and to close their eyes when measurements were performed. Thermal stimuli were transmitted through probes applied directly on the facial skin area. Participants were provided with a push button in their right hand to be used when the requested sensation was perceived. After giving instructions, just before the actual measurements, tests were demonstrated on a region outside the test area to acquaint the participant with the testing method. To increase reproducibility, the parameters were measured and calculated, as described in the DFNS's Investigator's Brochure^1,2^. - The thresholds for perceiving temperature changes were measured using a thermal cutaneous stimulator (QST.Lab, Strasbourg, France). - Mechanical thresholds were assessed by using a standardized set of twelve von Frey filaments (MRC Systems GmbH, Heidelberg, Germany) which are able to give stimuli of 0.25, 0.5, 1, 2, 4, 8, 16, 32, 64, 128, 256, and 512 mN when pressed perpendicularly to the skin. - The vibration detection threshold was determined by striking a tuning-fork (64 Hz) - Pressure pain thresholds were assessed with an algometer (Somedic SenseLab, Sösdala, Sweden)
Data format	Raw Analyzed Filtered
Description of data collection	Ten healthy Caucasian volunteers, five males and five females, aged between 18 and 35, were included in the study. Volunteers were recruited both among students and staff of the research institution as well as individuals outside the institution through a poster. None of the participants were subjected to a QST examination before. No monetary rewards were given. Participants were excluded in case of the following reasons: head and neck surgery within one year of the examination, trauma or neuropathies in the orofacial region, current infection in the orofacial region, psychiatric disorders, neurological and neurodegenerative disorders, inborn errors of metabolism, hyper- and hypothyroidism, diabetes, migraine, epilepsy, autoimmune diseases, dermatological conditions in the orofacial region, electrolyte imbalances and vitamin deficiencies, pregnancy and intake of analgesics or other medication in the last 48 hours.
Data source location	• Institution: Department of Oral and Maxillofacial Surgery, University Hospitals Leuven • City/Town/Region: Leuven • Country: Belgium
Data accessibility	Repository name: KU Leuven RDR [1] Data identification number: doi:10.48804/X7PJRD Direct URL to data: https://rdr.kuleuven.be/dataset.xhtml?persistentId=doi:10.48804/X7PJRD

## Value of the Data

• The data from this study can inform a larger clinical trial to generate a database of normative values for orofacial quantitative sensory testing (QST).
• The normative values can be useful for clinicians in evaluating and diagnosing orofacial somatosensory dysfunction.
• The data can be used by researchers to gain further insights into orofacial somatosensory function and to develop new experiments or studies.
• Clinicians and researchers working in the field of orofacial pain and sensory disorders are likely to benefit from these data.
• The findings may have broader implications for the fields of neuroscience, pain management, and sensory perception.

## 1 Objective

The main reasoning behind this dataset is to improve the assessment of orofacial somatosensory dysfunction using quantitative sensory testing (QST). QST is a non-invasive method of evaluating the sensitivity of various regions of the face and mouth innervated by the trigeminal nerve, which is responsible for sensory information from the head and face. While normal values have already been established for some parts of the face and mouth, there is still a lack of complete benchmark values for the entire innervation area of the trigeminal nerve.

This dataset aims to provide benchmark values and illustrate the differences in QST results across different anatomic subsets of the trigeminal nerve, both intraorally and extraorally, in healthy individuals. This information can be used to inform a larger clinical trial. Also, these normative values will be useful in medical practice, providing a benchmark for clinicians to evaluate and diagnose orofacial somatosensory dysfunction.

Overall, this dataset will improve the understanding of orofacial somatosensory function and establish reliable norms for QST, which can ultimately lead to better diagnosis and management of orofacial pain and other sensory disorders.

## 2 Data Description

**Fig. 1:**
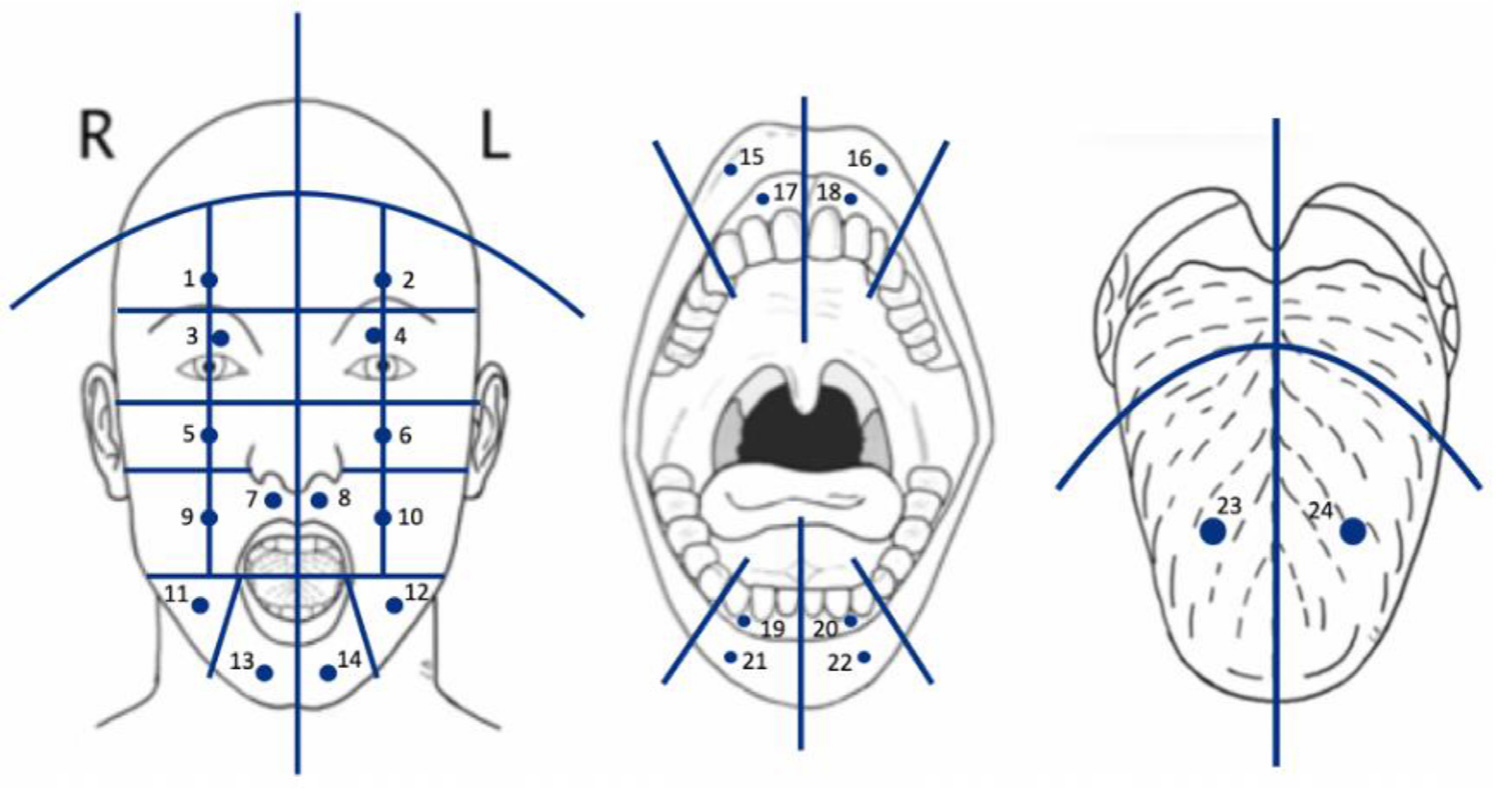
Test sites for orofacial quantitative sensory testing. Each number represents a test site. This number corresponds with the column “individual localization nr” in “raw data.xlsx”.

Raw data are publicly available under a Creative Commons CC-BY-NC-SA-4.0 license on the public KU Leuven RDR repository and titled “Orofacial quantitative sensory testing benchmark values”. The following files were included in the KU Leuven RDR repository: a plain text Readme.txt file and report.docx describing the materials and methods and included files. A list of used abbreviations in .xlsx format was included as well. For clarity this list is provided in Table 1 below. Also, the test sites and their respective test site numbers are illustrated in Fig. 1. A slide deck with QST patient instructions was uploaded to increase reproducibility (QST Patient Instructions.pptx).

**Table 1:** List of abbreviations.

Abbreviation	Description	Unit
2PD	Two-point discrimination	mm
CDT	Cold detection threshold	°C
WDT	Warmth detection threshold	°C
CPT	Cold pain threshold	°C
HPT	Heat pain threshold	°C
MDT	Mechanical detection threshold	mN
MPT	Mechanical pain threshold	mN
MPS	Mechanical pain sensitivity	mN
DMA	Dynamic mechanical allodynia	mN
WUR	Wind-up ratio	/100
VDT	Vibration detection threshold	/8
PPT	Pressure pain threshold	kPA

Next, we provided the raw data containing all the measurements in raw data.xlsx and summarized the mean and standard deviations for all participants stratified for the tested intra- and extraoral regions in summary table.xlsx. The column names are explained in Table 2.

**Table 2:** Column names and description presented in raw data.xlsx. Used abbreviations can be found in Table 1.

Column name	Description
Participant_asint Participant	Participant number
Regional nr_asint regional nr	Number corresponding with one test site. The corresponding test site can be found in column “trigeminal location”
individual localization nr	Number corresponding with the test sites illustrated in Fig. 1.
Side	Left or right sided test site
Trigeminal distribution	Trigeminal nerve distribution 1. ophthalmic distribution 2. maxillary distribution 3. mandibular distribution
Trigeminal region	Trigeminal nerve distribution but further stratified into extraoral and intraoral regions
Intraoral extraoral	Test site location intraoral or extraoral
2PD_difference_left_vs_right	Two-point discrimination absolute difference between left and right side
2PD_abs_difference_left_vs_right	Absolute value of 2PD_difference_left_vs_right

**Fig. 2:**
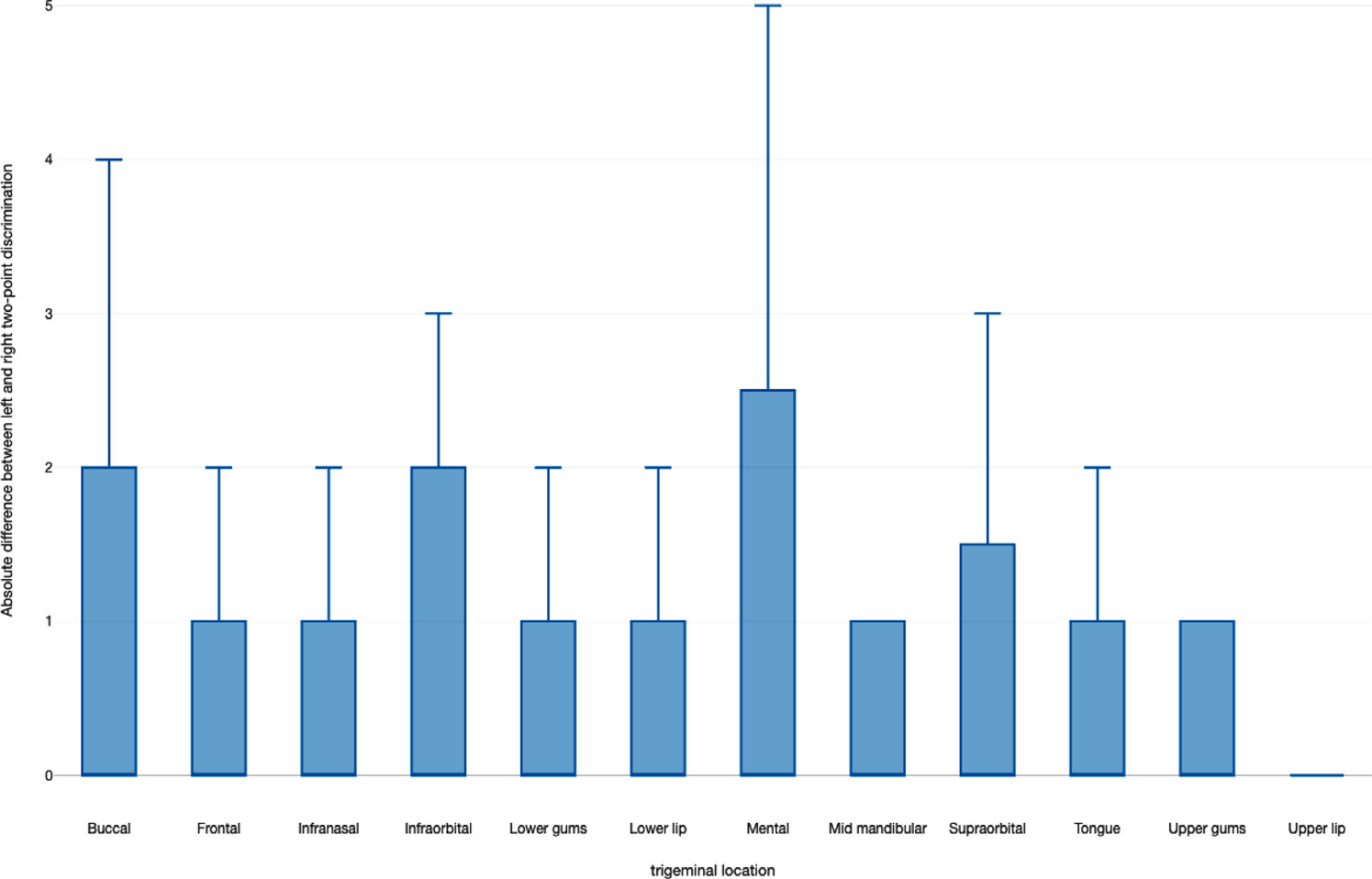
Boxplots with the absolute difference between two-point discriminations measured left versus right in millimeters and stratified according to the different test sites.

To aid clinicians in visualizing the absolute difference between the two-point discrimination test results obtained from the left and right side of the face or mouth, we have included a descriptive boxplot. The boxplot is graphically represented in Fig. 2.

Finally, a correlation study was done and represented in Correlations.xlsx. This file contains a comparative study to assess significant correlations between QST modalities and stratified by region.

## 3 Experimental Design, Materials and Methods

### 3.1 Participants

Ten healthy Caucasian volunteers, five males and five females, aged between 18 and 35, were included in the study. Volunteers were recruited both among students and staff of the research institution as well as individuals outside the institution through a poster. None of the participants were subjected to a quantitative sensory testing (QST) examination before. No monetary rewards were given. Participants were excluded in case of the following reasons: head and neck surgery within one year of the examination, trauma or neuropathies in the orofacial region, current infection in the orofacial region, psychiatric disorders, neurological and neurodegenerative disorders, inborn errors of metabolism, hyper- and hypothyroidism, diabetes, migraine, epilepsy, autoimmune diseases, dermatological conditions in the orofacial region, electrolyte imbalances and vitamin deficiencies, pregnancy and intake of analgesics or other medication in the last 48 hours. The study was approved by the Ethics Committee Research UZ Leuven (S63809) and all patients signed an informed consent form.

### 3.2 Study Design

This prospective study was performed between September and November 2020. All participants were tested at 14 extraoral and 10 intraoral regions on the face, distributed over the innervation area of the trigeminal nerve by the same trained examiner (HVB) who had previously completed a practical course and training session. The ophthalmic division (V1) constituted four test sites. The infraorbital and mandibular division each had ten test sites (Fig. 1). All instructions were given using a slide deck with prerecorded audio-instructions to improve standardization across sessions. The examination followed the DFNS protocol and the previously published modified intra-oral QST protocol [2,3].

### 3.3 Examination

For all tests the participants were seated in an examination chair, asked not to look at the computer screen and to close their eyes when measurements were performed. Thermal stimuli were transmitted through probes applied directly on the facial skin area. Participants were provided with a push button in their right hand to be used when the requested sensation was perceived. After giving instructions, just before the actual measurements, tests were demonstrated on a region outside the test area to acquaint the participant with the testing method. To increase reproducibility, the parameters were measured and calculated, as described in the DFNS's Investigator's Brochure [4].

### 3.4 Cold Detection Threshold (CDT) and Warmth Detection Threshold (WDT)

The thresholds for perceiving temperature changes were measured using a thermal cutaneous stimulator (QST.Lab, Strasbourg, France). The probe of this device, with a stimulation surface of 0.4 cm^2^ and a temperature range of 0°C to 50°C is gently pressed against the skin and, starting with a reference temperature adjusted to the skin temperature, cools down at a rate of 1°C/s. The participant uses the push-button upon perceiving a cold sensation, followed by an interval of four seconds, in which the temperature returns to the reference value. This process is repeated twice. The WDT is determined through a similar process, with the probe warming up, also at 1°C/s. The CDT and the WDT are the arithmetic means of the three detection thresholds, respectively. Thermal sensory limen and paradoxical heat sensations were not recorded in this study.

### 3.5 Cold Pain Threshold (CPT) and Heat Pain Threshold (HPT)

To determine the CPT and HPT, the probe cools down or warms up similarly as for the CDT and WDT (1°C/s). The participant uses the push-button only when the temperature is perceived as unpleasant. This is also done three times. CPT and HPT values are the arithmetic means of the thresholds reported.

### 3.6 Mechanical Detection Threshold (MDT)

MDT was assessed by using a standardized set of twelve von Frey filaments (MRC Systems GmbH, Heidelberg, Germany) which are able to give stimuli of 0.25, 0.5, 1, 2, 4, 8, 16, 32, 64, 128, 256, and 512 mN when pressed perpendicularly to the skin. The order of the stimuli is based on the modified “method of limits”. The test starts with the 16 mN filament, after which the intensity of the stimuli is gradually reduced until the participant reports no longer feeling the stimulus. This is the first subthreshold value, which is not registered. Next, stimuli with gradually increasing intensities are given, until a stimulus is perceived. The intensity at which stimulus perception returns is the first registered suprathreshold value. Thereafter, the stimulus intensity progressively decreases, allowing the measurement of the (second) subthreshold value. The final MDT is the geometric mean of the five subthresholds and suprathresholds determined by repeating this sequence four more times.

In case a participant does not feel the 16 mN filament at the beginning of the test, the 32 mN filament is tried, followed by the 64 mN filament, and so on, until a filament is perceived.

### 3.7 Mechanical Pain Threshold (MPT)

This test uses seven different weighed pinprick stimulators (MRC Systems GmbH, Heidelberg, Germany) of 8, 16, 32, 64, 128, 256, and 512 mN, all with a tip of 0.25 mm^2^. The modified “method of limits” is applied here as well. The participant reports for every stimulus whether it is perceived as solely a ‘dull feeling, or has a ‘sharp’, ‘prickly’ or ‘stingy’ component. A first stimulus of 8 mN is given, followed by stimuli of increasing intensities, until the participant reports a sharp sensation. This is the suprathreshold value. Next, stimuli with decreasing intensities are given to determine the subthreshold value at the intensity the sharp component is no longer perceived. The MPT is the geometric mean of the five pairs of suprathreshold and subthreshold values measured.

### 3.8 Mechanical Pain Sensitivity (MPS) and Dynamic Mechanical Allodynia (DMA)

The pinpricks are given in a non-consecutive order to determine the MPS. Participants score every stimulus from 0 to 100, with 0 meaning ‘no pain’ and 100 ‘worst pain ever’. This is done a total of five times, of which the geometrical mean forms the MPS.

The DMA is measured by using three different painless stimuli: (1) a soft brush (200-400 mN), (2) a Q-tip (100 mN), and (3) a tuft of cotton wool (3 mN). The geometric mean of the five repeats gives the intensity of DMA, if present.

The entire test consists of 50 stimuli. Although the pinpricks and painless stimuli are given in an apparently random order, this order is identical for all participants.

### 3.9 Wind-up Ratio (WUR)

WUR assesses the pain intensity over a given period of time when a stimulus is repeatedly delivered above a critical threshold. Using the 128 mN pinprick stimulator a single stimulus is provided first on the participant skin, which is scored from 0 to 100 by the participant. Next, ten consecutive stimuli are given with intervals of one second in an area of 1 cm². A score from 0 to 100 for the whole series is requested. This is then repeated four more times. The WUR is the ratio of the mean scores for the ten pinpricks and the mean scores for the single stimulus.

### 3.10 Vibration Detection Threshold (VDT)

The VDT is determined by striking a tuning-fork (64 Hz) and placing it on a bony protrusion in the test area. The participant has to report the instances of the first and last perceived vibrations. At the last moment, the thrill value is read off of the tuning-fork. This test is performed three times. The VDT is the arithmetic mean of these three values.

### 3.11 Pressure Pain Threshold (PPT)

The final test is the PPT. With a pressure algometer (Somedic SenseLab, Sösdala, Zweden) an increasing pressure (0.5 kPa/s) is exercised to a contact area of 1 mm² on a muscular part of the test area. The pressure increases until the participant reports it as painful. This test is done three times. The arithmetic mean of the three values obtained is the PPT.

### 3.12 Statistical Analysis

Mean values and standard deviations were calculated for each modality. Boxplots were created to show the distribution of the measurements per area. Comparisons were made between measurements in different regions. Pearson correlation tests evaluated pairs of QST modalities and were repeated by trigeminal region.

## Ethics Statements

The study was conducted according to the Declaration of Helsinki and approved by the Ethics Committee Research UZ Leuven (protocol number: S63809). All patients signed an informed consent form.

## CRediT authorship contribution statement

**Julie Adriaenssens:** Investigation, Writing – review & editing. **Hannah Vaesen Bentein:** Investigation, Writing – review & editing. **Reinhilde Jacobs:** Writing – review & editing, Supervision. **Constantinus Politis:** Writing – review & editing, Supervision. **Fréderic Van der Cruyssen:** Conceptualization, Methodology, Data curation, Writing – original draft, Writing – review & editing, Project administration.

## Declaration of Competing Interest

The authors declare that they have no known competing financial interests or personal relationships that could have appeared to influence the work reported in this paper.

## Data Availability

Orofacial quantitative sensory testing benchmark values (Original data) (Dataverse).
